# “Smart Optometry” phone-based application as a visual acuity testing tool among pediatric population

**DOI:** 10.15537/smj.2022.43.8.20220374

**Published:** 2022-08

**Authors:** Lina H. Raffa, Nasser T. Balbaid, Mariam M. Ageel

**Affiliations:** *From the Department of Ophthalmology (Raffa, Balbaid), King Abdulaziz University Hospital; and from Department of Ophthalmology (Ageel), Jeddah Eye Hospital, Jeddah, Kingdom of Saudi Arabia.*

**Keywords:** amblyopia, screening, smartphone, telemedicine, visual acuity

## Abstract

**Objectives:**

To evaluate the validity of a smartphone-based application for visual acuity (VA) testing in children and to compare parent and clinician-performed VA to standard VA assessment.

**Methods:**

A cohort of 100 children aged <18 years old was recruited. Subjects were randomly assigned to either start with conventional distance VA chart screening or the smartphone VA assessment twice by both the clinician and the caregiver if applicable. Near VA scores were assessed using the near vision E chart. Accuracy and reliability values were analyzed.

**Results:**

One hundred patients with an average age of 9.92 ± 3.0 years old were enrolled. The difference between conventional distance and application logMAR values was -0.023, and the difference between near vision and application logMAR values was -0.004. “Smart Optometry” had a sensitivity of 89.3% in detecting subnormal VA compared with conventional vision testing methods. Sensitivity in detecting subnormal VA was found to be higher in younger age groups up to 91.7% in comparison with the older age groups. The interclass correlation of application-measured VA scores by the caregivers and the clinician were 0.77 (95% CI; 0.67-0.83) using single measures and 0.87 (95%CI; 0.8-0.9) using average measures.

**Conclusion:**

“Smart Optometry” phone application was found to be an acceptable home-based VA testing tool with good inter-rater reliability for young children showing good sensitivity in detecting subnormal VA, but lower sensitivity in detecting amblyopia.

**T**he increasing incidence of permanent vision loss, driven by late detection of vision disorders such as amblyopia, is an alarming concern, wherein the limited and lack of early appropriate screening programs for young population are reported to be a contributing factor.^1^ However, visual disorders such as amblyopia can be treated if diagnosed early.^1^ The global prevalence of amblyopia is projected to affect 175.2 million people by 2030 and 221.9 million by 2040.^2^ Thus, it is crucial to implement and design vision-screening programs that are accessible to all people. Charts with various optotypes are considered the gold standard for measuring distance visual acuity (VA). However, mobile phone-based technologies have emerged during the coronavirus disease-19 (COVID-19) pandemic as a result of dependence on health digitalization and telemedicine during this period.^3^ Teleophthalmology services have been proven to effectively detect and manage patients with common eye conditions, including age-related macular degeneration, glaucoma and diabetic retinopathy.^4-6^

In terms of VA testing, a systematic review showed that a smartphone application called “peek acuity” was found to be highly correlated with the Snellen chart results, making it a promising screening tool in the pediatric population. However, it has limitations as it is only available on the Google Play platform.^7^ “Smart Optometry”, an application that contains a variety of tests, is available on 2 application stores (Google Play and Apple AppStore). It contains the same optotypes and decimals used in the clinic, and has been validated in the adult population.^8,9^ However, no studies have been carried out on children to test its accuracy and reliability.^9^

The study by Yeung et al^10^ showed that with the growing burden of vision impairment, vision-oriented smartphone applications will play a crucial role in care delivery.^10^ More specifically, the implementation of easily approachable and on-demand VA assessment tools is one of the main pillars in supporting teleophthalmology healthcare, especially in areas with a shortage of manpower and supplies.^10^

Determination of the validity of vision-oriented smartphone applications may help in the development of visual assessment tools that are more accessible to people.^11^ This study validates the use of “Smart Optometry” application use among children We aimed to compare the “Smart Optometry” application with traditional clinical examination results, to determine the capability of “Smart Optometry” in assessing VA with accuracy and to compare clinician and parent-performed VA results.

## Methods

In this non-interventional randomized cross-sectional study, 100 pediatric patients aged 5-16 years old, who visited the ophthalmology outpatient clinic between January and December 2021 were recruited. Patients with significant verbal or developmental delay and those with VA worse than 20/200 were excluded to ensure reliable results. Sufficient time was provided to explain the purpose, methods, and requirements of the study.

### Vision testing

Physical (in-person) tests were carried put to validate the “Smart Optometry” application with traditional office vision tests. A printed guide in the Arabic language was written by a linguist for both Google Play and Apple Store mobile users, which were handed out to the caregivers. The majority of the patients followed the written instructions with relative ease and minimal guidance. A free application named “Smart Optometry” (Smart Optometry, version 3.4 -full on Android and version 4.2 on Apple, Idrija, Slovenia) was downloaded from Apple Store onto iPhone 11 and Google Play onto Honor Play. “Smart Optometry” offers 15 different standardized tests including color vision, contrast sensitivity, amsler grids, and more. The application was installed on an iPhone 11 (screen size 6.1”) running iOS version 15 and HONOR PLAY (screen size 6.3”) with android version 9. Both phones possess high definition (HD) in-plane switching (IPS) liquid crystal displays (LCDs). The displays were automatically set at maximum brightness (100%). The section, “Visual Acuity+”, which provides an interactive VA assessment through Thumbling E with 9 levels of decimal values (0.1, 0.2, 0.25, 0.32, 0.4, 0.5, 0.63, 0.8, 1.0), was utilized. The phone was held 40 cm (15.5 inches) away with the aid of a 40 cm long string to measure the distance accurately. The patient was instructed to stay in the same position even if unable to visualize the letters.

Tumbling E optotypes were used in “Smart Optometry” to uniformly use the same optotypes across all the tests. ClearChart®2 Digital Acuity System (Reichert Technologies, New York, USA) device which is an electronic tumbling E chart was utilized for distance VA. The patient sat 6 m away from the electronic chart (20 feet). For near visual acuity, a near E chart, the Rosenbaum pocket vision screener, which assesses Tumbling E symbols until 20/100 VA, were used. Patients were able to follow up with a pointer. Subnormal VA was defined as a decrease in the age-based visual acuity cut-offs set by the American Academy of Pediatrics.^12^ For the analysis, VA >20/30 was used as the cut-off for comparison with the gold standard. Amblyopia was defined as ≥2-line difference between the eyes.

### Randomization and sequence

**Figure 1:**
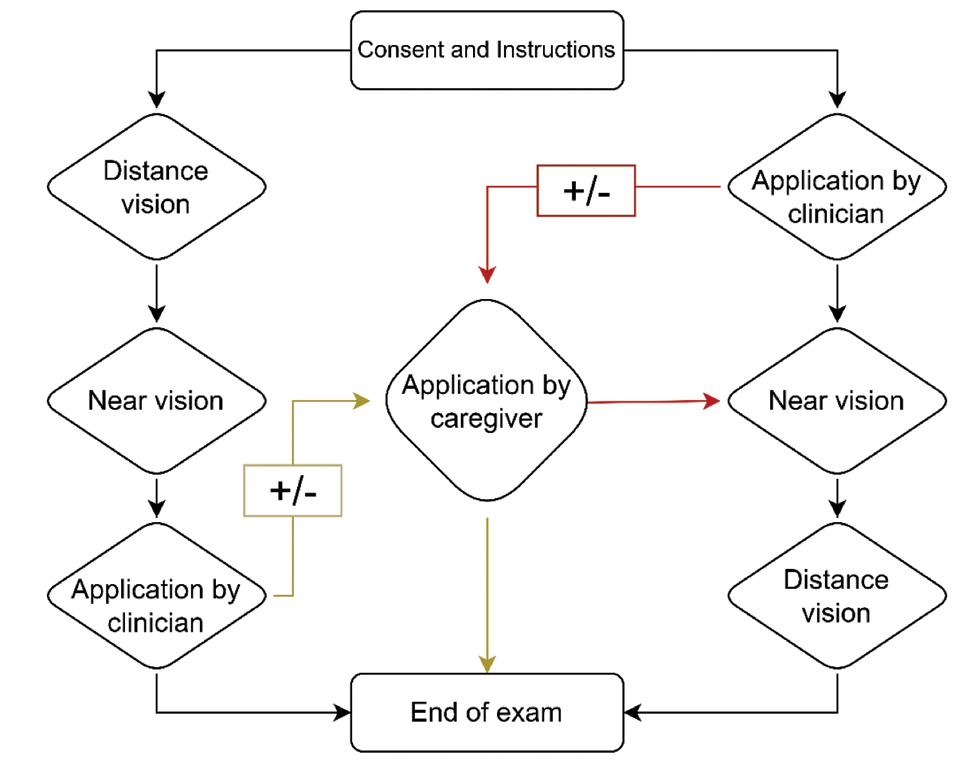
- Study flowchart visual acuity testing sequence.

The order of examination between the standard method and the smartphone was randomized for each patient to reduce changes in the test results due to fatigue and eye strain. The subjects were randomly assigned to start with the VA assessment in one of 2 sequences as shown in Figure 1. Using the application, the clinician encouraged the participants to point with their hands and swipe the touchscreen. For both the methods, VA was assessed monocularly, starting with the right eye, unless otherwise clinically indicated. A monocular occluder or adhesive occlusive patch was used to cover one eye, when the other eye was being tested. All participants were examined at the same clinic in a controlled environment. Standard VA assessment was performed by the same 2 clinicians (NB and MA).

The study was approved by the hospital’s local Institutional Review Board [Ref 42-21]. Appropriate informed consent (written and verbal) was obtained from the participant and the parent/guardian. This research was conducted in accordance with the Code of Ethics of the World Medical Association (Declaration of Helsinki 2013) for experiments involving humans. The authors also ensured the confidentiality of the patients by concealing any identifiers.

### Statistical analysis

The collected data were analyzed and presented using the Statistical Package for the Social Sciences, version 23 (IBM Corp., Armonk, N.Y., USA). Conversion to logMAR units for all VA measurement values was performed. Simple descriptive statistics were used to present study variables (categorical and nominal) as counts and percentages, while means and standard deviations were used to present continuous variables. Chi-square analysis was used to compare the relationship between categorical variables, while the independent t-test and one-way ANOVA were utilized to compare 2 group means and >2 groups. The correlation of variables (represented by means) was analyzed using Pearson’s correlation coefficient. A paired-samples t-test was then employed to compare the means of 2 variables (single group), to compute the differences between the values of 2 variables for each case, and to test whether the average differed from 0. To test proportional bias and the clinical agreement between 2 different test methods, Bland Altman analysis was used.^13^ The interclass correlation (ICC) was used to evaluate interobserver reliability between caregivers and clinicians. Furthermore, ICC showed an average measure of 0.8, indicated good reliability between raters. A *p*-value of <0.05 was set to reject the null hypothesis.

## Results

A total of 100 young patients with an average age of 9.92 ± 3.0 years old were enrolled in this study. More specifically, majority of them belonged to 5-8 (39%) years old and 9-12 (41%) years old age groups, followed by 21% belonging to 13-16 years old age group, respectively. The right eye was first examined in roughly two-thirds (65%) of the participants.

### Visual acuity measurement

All participants completed both the clinic-based (distance) and the application-based visual assessments. Three patients were unable to complete near VA testing because of examination fatigue. The median logMAR on conventional distance VA was 0.00 (interquartile range [IQR]=0-0.1), while the median logMAR for application-based visual acuity was 0 (IQR=0-0.2) by the clinician and 0 (IQR 0-0.1) by the caregiver.

### Visual acuity accuracy

**Figure 2:**
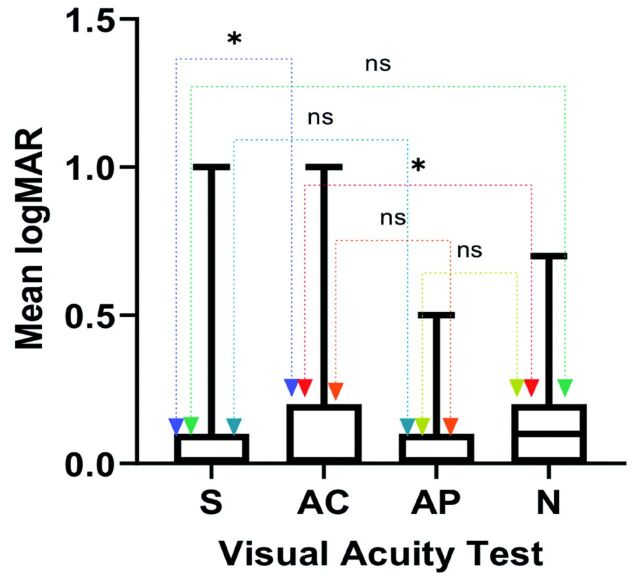
- Paired t-test between standard (S), application by clinician (AC), application by parent/caregiver (AP), and near (N) methods. NS: non-significant, ^*^indicates significance

**Figure 3:**
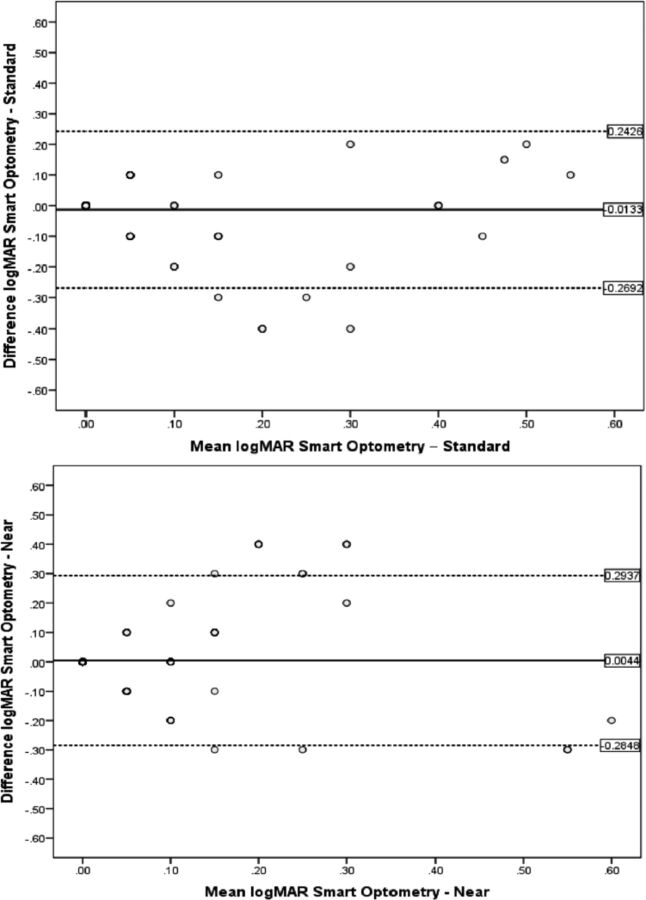
- Bland Altman plots for limits of agreement between visual acuity tested with **A**) Smart Optometry and standard Snellen chart and **B**) near vision chart.

A 6 pair comparison of 200 eyes of 100 patients was carried out in order to compare between VAs measured by the clinic-based tests (at near and distance) and the “Smart Optometry” application by both the caregivers and the clinician (Table 1 and Figure 2). Bland-Altman plots demonstrated no proportional bias between parent-performed application and standard distance (B=0.03, *p*=0.76) (Figure 3A) or near testing (B=0.06, *p*=0.644) (Figure 3B).

**Table 1:** - Six pair comparison of 200 eyes of 100 patients between visual acuity measured by clinic-based VA (at near and distance) and “Smart Optometry” application.

Paired samples statistics		n	Mean ± SD	Mean difference	95% CI of the difference		*P*-value
					Lower	Upper	
Pair 1	logMAR S	200	0.11 ± 0.2	-0.023	-0.041	-0.006	0.010^*^
	logMAR AC	200	0.13 ± 0.2				
Pair 2	logMAR S	94	0.06 ± 0.1	-0.013	-0.040	0.013	0.326
	logMAR AP	94	0.08 ± 0.1				
Pair 3	logMAR S	194	0.11 ± 0.2	-0.004	-0.021	0.013	0.634
	logMAR N	194	0.12 ± 0.2				
Pair 4	logMAR AC	94	0.09 ± 0.2	0.014	-0.007	0.034	0.184
	logMAR AP	94	0.08 ± 0.1				
Pair 5	logMAR AC	194	0.13 ± 0.2	0.018	0.002	0.034	0.024^*^
	logMAR N	194	0.12 ± 0.2				
Pair 6	logMAR AP	90	0.08 ± 0.1	0.004	-0.026	0.035	0.776
	logMAR N	90	0.08 ± 0.1				

^*^ Significant using paired samples test at <0.05 level. CI: confidence interval, VA: visual acuity, n: number

The mean difference between Snellen distance and “Smart Optometry” acuity was -0.023 LogMAR units or the equivalence of one letter (95% confidence interval [CI], -0.041 to -0.006), having the logMAR results being significantly higher (*p*=0.010) when tested using the “Smart Optometry” (0.13±0.2) compared to the standard Snellen procedure (0.11±0.2). A mean difference of 0.018 (95% CI, 0.002-0.034) was found between near vision and application logMAR, with the logMAR results being statistically higher (*p*=0.024) for the application (0.13±0.2) compared to the near chart VA (0.12±0.2). The “Smart Optometry” application, on average, underestimated by up to 2 letters at 95% CI when compared to the near VA. No statistically significant differences were observed between the other pairs (*p*<0.05).

### Amblyopia and subnormal VA detection

The capability of “Smart Optometry” to detect amblyopia and subnormal VA was evaluated. Using the conventional method, 28 patients suffered from subnormal VA in either eye and 12 from amblyopia. Overall, “Smart Optometry” revealed an 89.3% sensitivity and 69.4% specificity for identifying children with subnormal vision among all participants (Table 2).

**Table 2:** - Sensitivity and specificity of “Smart Optometry” application (clinician-performed) in detecting subnormal vision in 100 patients.

	Subnormal vision in the conventional				
Variables	Yes	No	Total	Predictive value	*P*-value
***Subnormal vision in the application***					
Yes	25	22	47	PPV (95% CI) 53.2%	<0.001^*^
No	3	50	53	NPV (95% CI) 94.3%	
Total	28	72	100		
Properties	Sensitivity (95% CI) 89.3%	Specificity (95% CI) 69.4%			

^*^ Significant using Chi-square test at <0.05 level. PPV: positive predictive value, NPV: negative predictive value

Among younger age groups (5-8 years old and 9-12 years old), sensitivity was found to be higher (90% and 91.7%) in comparison with the older age groups (13-16 years old) obtaining 80% sensitivity in detecting subnormal VA. However, specificity was found to be highest in the older age group, reaching up to 80%, with younger age groups ranging between 57.1% and 75.9%. With regards to amblyopia assessment, “Smart Optometry” application showed 58.3% sensitivity and 83% specificity in detecting amblyopia among all children (Table 3). Sensitivity and specificity for detecting amblyopia were lower in the younger age group (50% and 77%; 5-8 years old) compared to the older age group (64.8% and 84.5%; 9-12 years old). One-way ANOVA analysis revealed no differences in VA results among different age groups.

**Table 3:** - Sensitivity and specificity of “Smart Optometry” application (clinician-performed) in detecting amblyopia.

	Amblyopia by conventional test				*P*-value
Variables	Yes	No	Total	Predictive value	
***Amblyopia by application***					
Yes	7	15	78	PPV (95% CI) 31.8%	0.001^*^
No	5	73	22	NPV (95% CI) 93.6%	
Total	12	88	100		
Properties	Sensitivity (95% CI) 58.3%	Specificity (95% CI) 83.0%			

^*^ Significant using Chi-square test at <0.05 level. PPV: positive predictive value, NPV: negative predictive value, CI: confidence interval

### Interclass correlation

Of the 100 patients, 94 agreed to be tested twice by both clinicians and caregivers. Paired t-test showed no significant difference value of 0.014 logMAR units (95%CI; -0.0067 to 0.034; *p*=0.18) between the 2 methods. Furthermore, the interclass correlation of the application-measured VA scores by the caregivers and the clinician were 0.77 (95%CI; 0.67-0.83) using single measures, and 0.87 (95% CI; 0.8-0.9) using average measures

## Discussion

The COVID-19 pandemic has highlighted the importance of telemedicine. During this period, the pediatric clinic visits decreased, for both follow-ups and new visits.^14^ A significant proportion of patients either canceled their appointments or delayed them due to fear of acquiring infections, allowing the attention to be diverted to telemedicine services with the provision of uninterrupted healthcare.^15,16^ An increasing number of applications provide convenient and sufficient health care consultancy and tracking, which decreases the overcrowding of clinics and facilitates prioritization of healthcare delivery. In addition, the early detection of amblyopia may lead to more effective treatment. The best strategy to avoid permanent vision loss is to improve screening programs (especially for younger populations) and raise awareness of the problem. It is important to find a reliable, scalable, simple, and straight-forward VA testing programs that can be understood and utilized by all populations for any age, gender, or socioeconomic status, which does not require the patient’s physical attendance to the clinic.

Statistics showed an estimated 500 million phone users worldwide in the early 2000s.^17^ In 2012, more than 6 billion mobile phone subscribers have been estimated, and that 75% of global population had access to mobile communication.^18^ The evidently high rate of cellular phone users allows eHealth applications to be more appealing even in low-income settings.

Comparison of “Smart Optometry” application and Snellen chart standard method to test VA in this study showed one to 2 letters logMAR unit differences which reflects excellent validity. Although statistically significant, such minimal differences do not play a significant role in our daily clinical practice. Nonetheless, logMAR was found to be underestimated using the application when compared to the traditional distance chart. This is in accordance with the results of another study that explored iPad tablet computers as a means of VA testing tool.^19^ Near VA was found to be significantly different from the application. This is also in agreement with a study by Satgunam et al^9^ which also validated the “Smart Optometry” application, however in an adult population, and found near VA to be comparable to the presbyopic group.

Furthermore, the result of this current study revealed that the “Smart Optometry” application detected subnormal vision worse than 20/30 with sensitivity of 89.3% and specificity of 69.4%, and detected amblyopia with sensitivity of 58.3% but specificity of 83%. Its high sensitivity in detecting subnormal vision suggests that it can be utilized as a fairly accurate VA testing tool. In addition, the excellent 93.4% negative predictive value allowed to strongly infer that a child with no subnormal VA based on application test will most likely not suffer from subnormal VA testing when compared to the conventional methods. Our results are similar to a study carried out in Paraguay, which validated the use of peek acuity application to test VA among 6 and 16 year-old school aged children, showing 48% sensitivity and 83% specificity for identifying referable ocular disorders using a referral cut-off of visual acuity <20/40.^20^ Additionally, in agreement with another study carried in Kenya, peek acuity showed similar sensitivity and specificity of 77% and 91% for identifying children with visual acuity <20/40.^21^ However, in our study, the low sensitivity, specifically in the younger age group, in detecting amblyopia makes it less effective as a screening tool as it could fail to recognize children suffering from amblyopia who require further evaluation by an ophthalmologist which is the main objective of vision screening. Interclass correlation between different testers was found to be quite acceptable at 0.7 -0.8. In addition, the instructions were also reasonably clear to the caregivers. This could indicate that most people familiar with smart phones can manage this application. On the other hand, digital exclusion or poor eHealth literacy is an issue to consider even in high income countries which might be attributed to multiple factors, including gender, age, experience, education or culture.^22^

The strength of this study is that “Smart Optometry” is widely available and is a free application easily downloaded on Apple and Android smartphones, presenting 5 optotypes per line which is similar to the traditional vision testing chart rather than presenting single optotypes like “Peek acuity” application that simulates crowding.^23^ Second is the standardization of the examination settings, instructions to the caregivers, and the sequence of vision testing, which minimized biases. Third, peeking was also been controlled by using adhesive patches which has not been the case in other studies.^23^ Fourth, the lack of need to calibrate this application adds to its advantages over other applications.^9^

### Study limitations

The study include refusal of the subjects to participate and withdrawal from the study due to examination fatigue. Since recruitment was carried out in a pediatric ophthalmology clinic at a tertiary center, a higher prevalence of children with impaired vision was observed. In addition, is the small sample size, which was generally attributed to the these limitations. Studies involving large sample size and extended inclusion criteria (such as incorporation of patients with ocular pathologies) are recommended to further investigate the validity of the current results. Another limitation is the lack of masking of in-office visual acuity to both staff and caregivers. Moreover, the application performed sub optimally despite the tests were performed in a controlled setting. Less favorable results are anticipated in home-based testing of VA in children provided the variability in testing distance, illumination and propensity of children to peep. Thus, further validation would be needed to test it at home in less-than-ideal settings to identify potential challenges or benefits. Lastly, this study did not consider the feasibility of digital inclusion in our population whereby patients who need health care services might be the ones who are unable to use such an application or merely use it properly.

In conclusion, this study provided evidence on the validity of “Smart Optometry” application in testing visual acuity among pediatric patients when compared to the conventional approach, specifically estimating VA with sensitivity and fair accuracy in younger age groups. This study also emphasized the feasibility of using home-based health-related applications for preliminary VA assessment by non-health care professionals as parent-performed VA testing correlated well with clinic testing. This makes it a reasonable alternative for those with limited access to vision health care facilities. However, more easy to use and intuitive innovations with higher sensitivity are needed before this tool can be used in broader telehealth programs among young pediatric population prone to amblyopia. Increased public health awareness on amblyopia and training primary health care providers to test vision still plays a crucial role in preventing permanent blindness. Nonetheless, evidence shows that conventional VA screening by healthcare professionals is still the most accurate and cost-effective option for amblyopia screening.^24^
